# Fibrose pulmonaire révélant un lupus érythémateux systémique du sujet âgé

**DOI:** 10.11604/pamj.2014.18.311.5057

**Published:** 2014-08-20

**Authors:** Wafa Chebbi, Olfa Berriche

**Affiliations:** 1 Service de Médecine Interne, CHU Taher Sfar Mahdia, 5100 Mahdia, Tunisie

**Keywords:** Fibrose pulmonaire, lupus érythémateux systémique, sujet âgé, pulmonary fibrosis, systemic lupus erythematosus, elderly

## Image en medicine

La fibrose pulmonaire est une manifestation rare du lupus érythémateux systémique (3 à 9%) et exceptionnellement révélatrice. Son étiopathogénie demeure mal élucidée. Sa présentation clinique et radiologique ne semble pas être différente de celle observée au cours des autres connectivites. Elle s'accompagne souvent d'atteintes multiviscérales. Nous rapportons l'observation d'un patient âgé de 82 ans, sans antécédents pathologiques, hospitalisé pour une dyspnée stade III, une toux sèche et une asthénie, le tout évoluant depuis 3 mois. A l'anamnèse, il y avait une notion de photosensibilité et des polyarthralgies inflammatoires des grosses articulations. Le bilan biologique montrait un syndrome inflammatoire (vitesse de sédimentation à 110 à la première heure, CRP à 16 mg/dl, fibrinogène à 6,2 g /l) et une leucopénie à 1800 éléments mm^3^. Le bilan immunologique montrait des anticorps anti nucléaires à 1/1600 avec des anticorps anti-DNA positifs. Une échographie cardiaque montrait un épanchement péricardique de moyenne abondance. Le diagnostic d'un lupus érythémateux systémique était retenu devant la présence de 5 critères de l'ACR (photosensibilité, péricardite, lymphopénie, anticorps anti-nucléaires et anticorps anti-DNA positifs). La radiographie de thorax montrait un syndrome interstitiel bilatéral. Le scanner thoracique révélait de multiples images micro-kystiques réalisant un aspect en rayon de miel avec épaississement des lignées septales et présence par ailleurs de micronodules évoquant un aspect de fibrose interstitielle diffuse bilatérale. A l'exploration fonctionnelle respiratoire, il existait un syndrome restrictif. Le patient était mis sous corticothérapie à la dose de 1 mg/kg /j avec une évolution stable.

**Figure 1:**
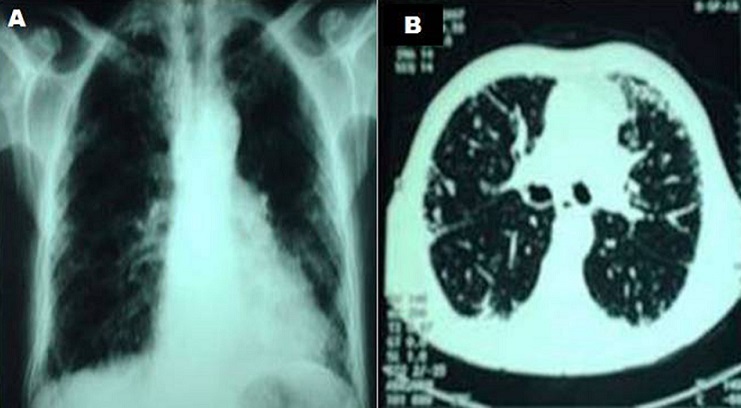
A) Radiographie de thorax: syndrome interstitiel bilatéral; B) TDM thoracique en coupe axiale: multiples images micro-kystiques réalisant un aspect en rayon de miel avec épaississement des lignées septales et présence par ailleurs de micronodules évoquant un aspect de fibrose interstitielle diffuse bilatérale

